# Antiviral Profile of Brown and Red Seaweed Polysaccharides Against Hepatitis C Virus

**Published:** 2016

**Authors:** Saly F. Gheda, Hala I. El-Adawi, Nehal M. EL-Deeb

**Affiliations:** a *Botany Department, Faculty of Science, Tanta University, Tanta, Egypt. *; b *Medical Biotechnology Department, Genetic Engineering & Biotechnology Research Institute, Alexandria, Egypt. *; c *Biopharmaceutical Product Research Department, Genetic Engineering & Biotechnology Research Institute, Alexandria, Egypt.*

**Keywords:** Seaweeds, polysaccharides, hepatitis C virus, free radicals, PCR

## Abstract

Hepatitis C virus (HCV) has infected 3% of the population worldwide and 20% of the population in Egypt. HCV infection can lead to hepatocellular carcinoma and death. The presently available treatment with interferon plus ribavirin, has limited benefits due to adverse side effects. Seaweeds have become a major source of new compounds to treat viral diseases. This work aimed to study the effect of four species of seaweeds as anti- HCV. The inhibition of lipid peroxidation was measured by evaluating the ability of seaweed extracts to scavenge the free radicals. The HepG2 cells were infected with the HCV and treated with each seaweed polysaccharide. Inhibition of viral replication was detected using the Real Time PCR (RT) qPCR. To explain the mode of the seaweed action on HCV, three modes of virus infections and seaweed polysaccharide treatments were applied. All treatments had the ability to inhibit the HCV with priority to *Laurencia obtusa* (82.36%), while the potentiality to scavenge the free radicals reached up to 81.5% with the *Sargassum* *vulgare*. Seaweed polysaccharide extracts may be helpful in exploring further gateways for antiviral therapy against HCV.

## Introduction

Hepatitis C virus (HCV) is an etiological agent of non-A and non B hepatitis infecting more than 170 million people worldwide (1). Infection with HCV leads to chronic liver disease in the majority of cases that can progress further to hepatocellular carcinoma (2). Egypt has the highest HCV prevalence worldwide. The most prevalent genotype HCV in Egypt is 4 a. The current therapeutic protocol for HCV infection consists mainly of interferon in combination with ribavirin that usually accompanies with strong side effects and moderate successful rate (3). This treatment is expensive and relatively toxic. Moreover, response to IFN- α/ribavirin combination therapy showed to have great variation among patients infected with different HCV genotypes. One of the most important reasons for the lack of success in developing antiviral drugs is due to the nature of the infectious viral agents, which totally depend upon the cell they infect for multiplication and survival. Thus, the compounds that may cause the death of viruses also are very likely to injure the host cell that harbours them (4). Efforts have been made to evaluate the antiviral activity of natural products, including those from algae, in order to characterize new compounds which inhibit virus replication and /or treat viral infection (5). The Egyptian coast is particularly rich in algal biodiversity and constitutes a reserve of species of considerable economic, social and ecologic potential. Nothing is known about the anti-HCV activities of extracts of brown and red seaweeds that collected from the Mediterrian coast of Egypt. Screening assays of the antiviral activity of extracts from some seaweeds have led to the identification of a number of sulfated polysaccharides having potent inhibitory effects against herpes simplex virus (HSV), human cytomegalovirus (HCMV), human immunodeficiency virus type-1 (HIV-1), respiratory syncytial virus (RSV) and influenza virus (5). Thus, the antiviral potential of polysaccharides extracted from seaweeds is of considerable interest (6). Many studies focused on the antiviral activity of seaweeds on many virus types. However, test of the antiviral activity of seaweeds against Hepatitis C virus (HCV) is lacking.

Herein, we investigate *in-vitro* the antiviral activity of polysaccharides from four seaweed species ((*Pterocladia capillacea* and *Laurencia obtusa* (Rhodophyta), *Sargassum vulgare *and *Padina pavonica* (Phaeophyta) against HCV. The aim will extend to evaluate the safety of seaweed polysaccharides in the treatment of HCV.

## Experimental

*Collection of Seaweeds*

 The four studied seaweeds were collected from the rocky bay of Abu Qir, Alexandria, Egypt in July 2008. They were washed thoroughly with tap water, dried in room temperature and pulverized in a blender. Identification of the seaweeds was carried out according to Aleem (7).

*Extraction of the crude seaweed polysaccharides*

The seaweed powder was boiled in distilled water (1:5 w/v) at 100 °C for 2 h using a reflux condenser under reduced pressure. The hot extract was filtered with a nylon mesh bag (pore size 24 μM) and sequentially filtered with 0.45 μM Millipore filters, then subjected to freeze-drying (8). Identification of phycocolloids extracted from seaweed samples was carried out by the first author with others using Vibrational Spectroscopy Fourier transform infra-red with attenuated total reflectance (FTIR-ATR) (9).

*Cell culture*

In the last few years, a number of cell culture systems have been developed that support reliable and efficient progression of HCV virus. Several human hepatocyte cell lines were analyzed. The hepatocellular carcinoma HepG2 cell line was found to be one of the most susceptible cell to HCV infection (10). 

HepG2 cells were washed twice with RPMI1640 media supplemented with 200μM L-glutamine and 25μM HEPES buffer; N-[2-hydroxyethyl] piperazine-N`- [2-ethanesulphonic acid] (all chemicals and media, Cambrex). The cells were suspended at 2×10 ^5^ cells/ mL in RPMI culture media (RPMI supplemented media, 10% fetal bovine serum (FBS); Gibco-BRL). The cells were left to adhere on the polystyrene 6 well plates for 4 h in an incubator (37 °C, 5% CO_2_, 95% humidity). The cells were washed twice from debris and dead cells by using RPMI supplemented media.

*Human blood lymphocytes separation*

Peripheral blood cells (PBMCs) were isolated, as reported by Lohr *et al*. (11). Briefly, peripheral blood samples were diluted with 5 volumes of a freshly prepared RBC lysis buffer (38.8 mmol/L NH_4_Cl, 2.5 mmol/L K_2_HCO_3_, 1 mmol/L EDTA, pH 8.0), incubated at room temperature for 10 min and centrifuged at 1500 rpm for 5 min. The nucleated cells were precipitated in the bottom of the tube.

*Cytotoxicity assays*

Using the human separated PBMCs and HepG2, cytotoxicity was evaluated by incubating cellular suspensions (2.5 × 10^5^ cells/mL, and 10^5^ cells/mL, respectively) in 24-well microliter plates and cultured for 48 h at 37 °C, 5% CO_2_. The culture was then refreshed with new RPMI-1640 supplemented medium containing serial dilutions of seaweed extracts (concentration from 2 to 20 mg/mL, 4 wells per concentration) and incubated for 90 min and washed three times with 1 mL of PBS buffer. Cytotoxicity by cell viability was measured using the neutral red dye method from Le Contel *et al.* (12). The stain intensity was assayed using the automated ELISA microplate reader adjusted at 540 nm (reference filters 620 nm). The cytotoxicity percentage values = [(OD) C − (OD)T/(OD)C] × 100. (OD)C and (OD)T, where the OD values are of the untreated and treated cells, respectively (13).

*First approach of the antiviral activity mode of seaweed polysaccharide extracts on HCV*

The inhibitory effect of the applied seaweed extracts on HCV was recorded on semi-confluent monolayers of human PBMCs (2.5×10^5^ cells/mL) and HepG2 (10^5^ cells/mL) infected with HCV (8.3×10^6^ copies/mL, genotype 4a), under different assays. 

In assay 1 (pre-treated or neutralization assay) the seaweed extracts of the nontoxic concentrations in 50 mL of RPMI-1640 supplemented medium were put in contact with the virus (infected serum) for 1 h at 4 ºC. Then, the host cells (PBMCs: 2.5×10^5^ cells/mL, or HepG2: 10^5^ cells/mL) were infected with the treated virus and incubated for 90 min at 37 °C, 5% CO_2_. The cells were washed three times with 1 mL of PBS and further cultured for 7 days at 37 °C, 5% CO_2_, followed by total RNA extraction. 

In assay II (Co-treated), the seaweed extracts of the nontoxic concentrations in 50 mL of RPMI-1640 supplemented medium were added simultaneously with the virus (infected serum) to the host cells (PBMCs: 2.5×10^5^ cells/mL, or HepG2: 10^5^ cells/mL), and after 90 min of incubation, infected cells were washed three times with 1 mL of PBS and further cultured for 7 days at 37 °C, 5% CO_2_, followed by total RNA extraction. 

In assay III (Post-treated), the infected serum (8.3×10^6^ copies/mL, genotype 4a), were added to the host cells and incubated for 90 min at 37 °C, 5% CO_2_. 

The cells were washed three times with PBS. The seaweed extracts of the nontoxic concentrations in 50 mL of RPMI-1640 supplemented medium were added and cultured for 7 days at 37 °C, 5% CO_2_, followed by total RNA extraction.

*RNA extraction from PBMCs and HepG2 cells*

RNA was isolated from the PBMCs and HepG2 cells as described by Lohr *et al.* (11). Briefly, the cells were precipitated and washed in the same buffer to remove adherent viral particles before lysis in 4 mol/L guanidinium isothiocyanate containing 25 mmol/L sodium citrate, 0.5% sarcosyl and 0.1 mol/L-mercaptoethanol and 100 μL sodium acetate. The lysed cells were centrifuged at 12000 rpm for 10 min at 4 ºC. The aqueous layer was collected and mixed with an equal volume of isopropanol. After incubation at 20 ºC overnight, RNA was precipitated by centrifugation at 12000 rpm for 30 min at 4 ºC and the precipitate RNA was washed twice with 70% ethanol.

*PCR and real time PCR of genomic and anti-genomic RNA strands of HCV*

Reverse transcription-nested PCR was carried out according to Lohr et al. (11), with some modifications. Retrotranscription was performed in 25 μL reaction mixture containing 20 U of AMV reverse transcriptase (Clontech, USA) with either 400 ng of total HepG2 or PBMCs cells RNA, 40 U of RNAsin (Clontech, USA), a final concentration of 0.2 mmol/L from each dNTP (Promega, Madison, WI, USA) and 50 pmol of the reverse primer 1CH (for plus strand) or 50 pmol of the forward primer 2CH (for minus strand). The reaction was incubated at 42 ºC for 60 min and denatured at 98 ºC for 10 min. Amplification of the highly conserved 5′-UTR sequences was done using two roundsPCR with two pairs of nested primers. First round amplification was done in 50 μL reaction mixture, containing 50 pmol from each of 2CH forward primer and P2 reverse primer, 0.2 mmol/L from each dNTP, 10 μL from RT reaction mixture as template and 2 U of Taq DNA polymerase (Promega, USA) in a 1× buffer supplied with the enzyme. The thermal cycling protocol was as follows: 1 min at 94 ºC, 1 min at 55 ºC and 1 min at 72 ºC for 30 cycles. The second round amplification was done similar to the first one, except for use of the nested reverse primer D2 and forward primer F2 at 50 pmol each. A fragment of 174 bp was identified in the positive samples. Primer sequences were as follows: 1CH: 5′-ggtgcacggtctacgagacctc-3′, 2CH: 5′-aactactgtcttcacgcagaa-3′, P2: 5′- tgctcatggtgcacggtcta-3′, D2: 5′-actcggctagcagtctcgcg- 3′ and F2: 5′-gtgcagcctccaggaccc-3′. To control false detection of negative-strand HCV RNA and known variations in PCR efficiency, specific control assays and rigorous standardization of the reaction were employed: (1) cDNA synthesis without RNA templates to exclude product contamination; (2) cDNA synthesis without RTase to exclude Taq polymerase RTase activity; (3) cDNAsynthesis and PCR step done with only the reverse or forward primer to confirm no contamination from mixed primers. These controls were consistently negative. In addition, cDNA synthesis was carried out using only one primer present followed by heat inactivation of RTase activity at 95 ºC for 1 h, in an attempt to diminish false detection of negative-strand prior to the addition of the second primer. Finally, the RT-PCR was done to the final PCR product based on the SYBR Green I dye and LightCycler fluorimeter. Amplicon synthesis was monitored continuously by SYBR Green I dye binding to double stranded DNA during PCR of the 5′ HCV non-coding (NC) region. Specificity was verified by amplicon melting temperatures. An external standard curve was constructed with serial 10 fold dilutions of a modified synthetic HCV 5′ NC RNA (14).

*Strand-Specific RT-qPCR*

The real time quantitative (RTq) PCR was done to the final PCR product based on the SYBR Green I dye and Light Cycler fluorimeter using a standard HCV infected serum samples. Amplicon synthesis was monitored continuously by SYBR Green I dye binding to double stranded DNA during PCR of the 5′ HCV non-coding (NC) region. Specificity was verified by amplicon melting temperatures. An external standard curve was constructed with serial 10 fold dilutions of a modified synthetic HCV 5′ NC RNA (15), The RT-qPCR step was followed by first round PCR using the 2CH and P2 primers in 50 µL reaction containing 20 µL cDNA, 50 pmol from each of 2CH forward primer and P2 reverse primer and 12.5 µL master mix SYBR Green, The thermal cycling protocol was as follows: 30 cycles of 1 min each at 94, 55 and 72 ºC, with the final extension done at 72 ºC for 5 min.

*Lipid peroxidation assays*

HepG2 cells (1×10^5 ^cells) were seeded onto 6 well plates and treated with infected serum in a co treated model. After the treatment, cells were washed twice in prewarmed PBS (pH-7.2) and scraped into 2 mL PBS, cell suspension were used for thiobarbituric acid reacting substances (TBARS) assay (16). After 1 h of incubation of cell suspension, 1.0 mL of 5% TCA and 1.0 mL of 0.67% TBA were added in samples. The reaction mixture from the vial was transferred to the tube and centrifuged at 3500 rpm for 15 min. The supernatant was transferred to another tube and placed in a boiling water bath for 10 min. Thereafter, the test tubes were cooled and the absorbance of the color was read at 532 nm. The rate of lipid peroxidation was expressed as nmol of malonaldehyde (MDA) formed.

The lipid peroxidation inhibition rate % = [1 - (A1 -A2)/A0] × 100

Where, A0 is the absorbance of the control (without sample), A1 is the absorbance of the sample addition and A2 is the absorbance without cell line homogenate.

*Statistical Methodology*

Analysis of variance (one-way ANOVA) was used to identify statistically significant differences in peripheral blood mononuclear cells and HepG2 and different concentrations of seaweeds extracts. Significant diffrences means among the seaweed extracts were identified using the least significant differences (LSD) test at P<0.05. ANOVAs. All statistical analyses were performed using SPSS 15.0 software SPSS (2006).

**Figure 1:**
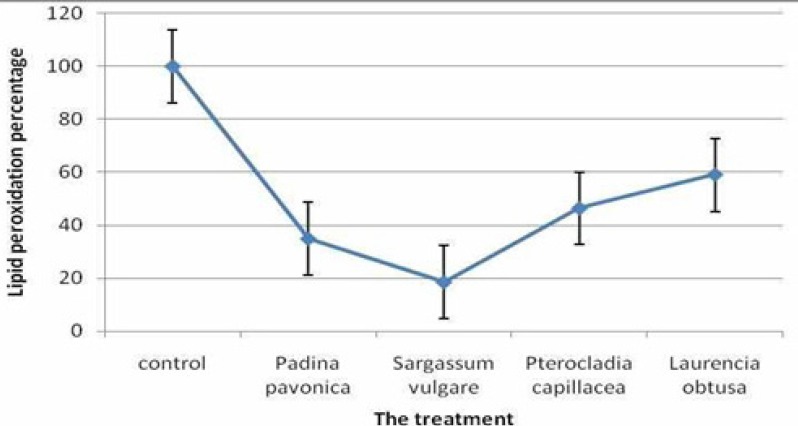
Lipid peroxidation inhibition of polysaccharide extract of *Padina pavonica,* *Sargassum vulgare, Pterocladia capillacea* and *Laurencia obtus*

**Figure 2:**
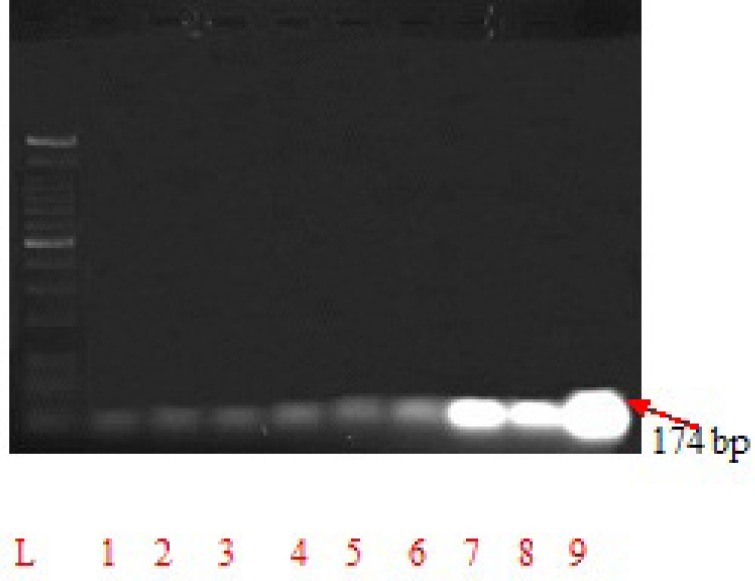
Inhibition of viral replication by *Padina pavonica,* *Sargassum vulgare, Pterocladia capillacea* and *Laurencia obtuse* polysaccharide extracts*. ***Lane L, **DNA ladder**, Lane 1,*** Padina pavonica *co-treatment, **Lane** **2, ***Sargassum vulgare* co-treatment, **Lane 3, ***Pterocladia capillacea* co- treatment and **Lane 4, ***Laurencia* *obtuse* co-treatment.** Lane 5,*** Padina pavonica *pre-treatment, **Lane 6, ***Sargassum vulgare* pre-treatment, **Lane** **7, ***Pterocladia capillacea* pre- treatment and **Lane 8, ***Laurencia obtuse* pre-treatment.** Lane 9, **amplified 174 bp of HCV from a positive control sample

**Table 1:** Mean± SD of cytotoxicity of seaweed extracts on peripheral blood mononuclear cells (PBMC). F-values represent the one way ANOVA, df=3. Means in the same columns followed by different letters are significantly different at P < 0.05

**Treatment concentrations mg/mL**	**Cytotoxicity percentage on PBMC**				**F- value**
	*Padina pavonica*	*Sargassum vulgare*	*Pterocladia capillacea*	*Laurencia obtusa*	
**2**	7.9±0.37	1.2±0.17	5.0±0.28	3.8±0.40	4.7^ns^
**4**	8.1±0.57ad	2.7±0.34	5.5±0.36	6.1±0.29da	9.2^ns^
**6**	8.4±0.33ad	4.1±0.29	8.2±0.13	9.8±0.39da	20.2*
**8**	10.7±0.39abcd	5.2±0.21bacd	13.5±0.30cabd	10.4±0.30dacb	55.6***
**10**	14.0±0.32 abcd	12.2±0.22ba	15.1±0.30cd	16.4±0.21da	30.6**
**12**	20.3±0.21	14.6±0.46	18.1±0.31	18.7±0.5	5.5^ns^
**14**	22.0±0.36	19.3±0.18	24.3±0.20	26.6±0.28	0.88^ns^
**16**	23.7±0.36	21.6±0.31	26.3±0.24	31.1±0.31	15.5^ns^

* P<0.05,

** P<0.01,

*** P<0.001 and ns = not significant (i.e. P>0.05).

**Table 2:** Mean± SD of cytotoxicity of seaweed extracts on (HepG2) cells. F-values represent the one way ANOVA, df=3. Means in the same columns followed by different letters are significantly different at P < 0.05

**Treatment concentrations mg/mL**	**Cytotoxicity percentage on HepG2**				**F-value**
	*Padina pavonica*	*Sargassum vulgare*	*Pterocladia capillacea*	*Laurencia obtusa*	
**2**	10.8±0.39ad	5.6±0.32	9.1±0.44	3.2±0.13da	17.8*
**4**	18.5±0.29abcd	9.4±0.32bacd	11.9±046cabd	7.7±0.37dabc	45.9**
**6**	43.6±0.33	59.2±0.34	15.9±0.44	20.4±0.23	36.8^ns^
**8**	44.6±0.35	59.4±0.44	28.2±0.21	51.0±0.27	85.9^ ns^
**10**	46.6±0.33	73.8±0.43	37.7±0.45	62.0±0.27	79.2^ ns^
**12**	69.1±0.57	74.2±0.14	69.2±0.22	69.8±0.50	60.7^ ns^
**14**	72.7±0.35	77.8±0.40	75.3±0.22	76.4±0.31	88.7^ ns^
**16**	76.6±0.32	83.9±0.47	76.1±0.29	89.9±0.49	80.1^ ns^

* P<0.05,

** P<0.01 and ns = not significant (i.e. P>0.05).

**Table 3:** Inhibiting effect on the HCV (copies/mL) of the polysaccharide of different seaweeds *Padina pavonica *, *Sargassum vulgare*, *Pterocladia capillacea* and *Laurencia obtusa*. The maximum inhibition value for each treatment of each seaweed was underlined

**Item**		**CT**	**Virus concentrations copies / mL**	**Inhibition percentage (%)**
**Control**		28.06	1.13×10^10^	-
***Padina pavonica***	pre-treated	12.22	3.4×10^9^	56.4
	co-treated	5.41	6.7×10^6^	80.7
	post-treated	16.23	5.4×10^9^	42.15
***Sargassum vulgare***	pre-treated	10.03	2.3×10^9^	64.25
	co-treated	5.16	6.3×10^6^	81.61
	post-treated	20.03	7.3×10^9^	28.62
***Pterocladia capillacea***	pre-treated	13.07	3.8×10^9^	53.42
	co-treated	8.46	1.5×10^9^	69.85
	post-treated	19.06	6.8×10^9^	32.07
***Laurencia obtusa***	pre-treated	10.03	2.3×10^9^	64.25
	co-treated	4.95	6.00×10^6^	82.36
	post-treated	15.43	5.01×10^9^	45.01

## Results

*Cytotoxicity*

Cytotoxicity test on PBMC was carried out to evaluate the safety of the seaweed treatments on the normal cells. We found that *Sargassum vulgare* extract was slightly safer than the others. The concentration 8 mg/mL was highly significant (P**<**0.001) on PBMC, while the concentrations of 10 mg/mL and 6 mg/mL were significant (P**<**0.01 and P**<**0.05 respectively). However, concentration 4 mg. ml^-1 ^statistically was not significant (p˃0.05), but the least significant difference (LSD) was 4.85. 

With an overview on the results, the non toxic doses (recommended) of *Padina pavonica*, *Sargassum vulgare*, *Pterocladia capillace* and *Laurencia obtusa* extracts were 8, 9, 7 and 8% mg/mL, respectively (Table 1.). On the other hand, concerning the cytotoxicity of the seaweed extracts on the HepG2 cancerous cell line, only the concentrations 4 mg/mL and 2 mg/mL were found significant (P**<**0.01 and P**<**0.05 respectively). The non toxic treatment concentrations were 1, 4, 3, and 5 mg/mL for extracts of* Padina pavonica, Sargassum vulgare*, *Pterocladia capillace *and* Laurencia obtuse*, respectively (Table 2.). 

*Inhibition of lipid peroxidation*

The ability of the algal extracts to protect liver from lipid peroxidation depends mainly on their ability to scavenge the induced free radical after HCV infection. The studied seaweed extracts protected the HepG2 cells from lipid peroxidation with a wide range of inhibition percentages. The maximum lipid peroxidation inhibition (81.54%) was attained by the co-treated HepG2 cell line with *Sargassum vulgare* extracts. In addition, the lipid peroxidation inhibition of the other treatments was 65.16, 53.63 and 40.93% for the *Padina pavonica,* *Pterocladia capillacea* and *Laurencia obtuse* extracts, respectively (Figure 1.).

*Antiviral effect of seaweeds extracts*

Inhibition of viral replication was detected by amplification of the viral RNA segments using PCR and RT-PCR techniques. The obtained results were by analogy with a standard curve of different known virus concentrations in a positive control infected cells. The tested extract is considered to be active when it is capable of inhibiting the viral replication inside the HCV-infected cells, as evidenced by the inhibition of virus concentration (copies/ ml) compared with the positive control (Figure 2.)**. **We detected that all extracts of the study have inhibitory effect with priority to co-treatment mode (Table 3.). *Laurencia obtuse* extract showed the highest antiviral potentialities by reducing viral concentration from 1.13×10^10 ^to 6.00×10^6^ copies /mL, with inhibition of 82.36%. Extracts of *Sargassum vulgare*, *Padina pavonica* and *Pterocladia capillacea*, respectively are less antiviral. They have reduction of viral concentration to 6.3×10^6^, 6.7×10^6^ and 1.5×10^9^ copies /mL, respectively and inhibitions of 81.61, 80.7 and 69.85%, respectively (Table 3. and Figure 2.).

## Discussion

The carried out cytotoxicity test on PBMC and HepG2 of the cancerous cell lines revealed that application of the sulphated polysaccharides from all studied seaweeds are safe at some of their concentrations. These results are consistent with those obtained for the isolated compounds from some plants. Wang *et al.* (16) identified pheophytin from ethanol-soluble fraction of *Lonicera hypoglauca* and applied it as anti HCV. This application caused an elicited dose-dependent inhibition of HCV viral proteins and RNA expression in both replicon cells and cell culture infectious system. Also, Haid *et al.* (17) found that flavonid (Ladanein) isolated from *Marrubium peregrinum* L (Lamiaceae) is effective against all major HCV genotypes. No or very low toxic effect on the treated cells was detected. 

In contrary, the current approved chemical therapies with interferon in combination with ribavirin used as anti-HCV are only partially effective and treatment is accompanied by serious side effects and moderate successful rate (3). In addition the usage of these chemical treatments is highly expensive. Seaweeds in Egypt coasts are widely spread, that make their usage as a source for anti-HCV compounds is not expensive in comparison with the chemical treatments. 

Sulfated polysaccharides including fucoidan are reported to inhibit the growth of various enveloped viruses (18). Fucoidan is thought to inhibit virus adsorption to the cell surface by binding to it, with subsequent prevention of cell infection (19). In addition, fucoidan interacts directly with the envelope glycoprotein on dengue virus type (20). Pereira *et al.* (9) described polysaccharides in *Padina pavonica *and *Sargassum vulgare* (the studied brown seaweeds). They showed that polysaccharide extracts from these seaweeds are mainly fucoidan, on which the *S. vulgare* fucoidan content exceeds that of the *P. pavonica* fucoidan. These polysaccharide compositions have been reflected on the potential effect of the extract as anti- HCV, where, the inhibitory effect of *S. vulgare* extract was higher than that of *P. pavonica *in the pre-treated and co-treated modes (Table 3.). The co-treated modes gave the highest inhibition percentage of the virus. This may be due to the seaweed extracts added simultaneously with the virus and the host cells, so they can bind immediately with the cell surface of host cell and prevent virus adsorption and penetration. Thus, the fucoidan may inhibit virus adsorption to the cell surface by binding to it. At the post-treated mode, the inhibition of the *P. pavonica* extract (42.15%) was much higher than that of the *S. vulgare* extract (28.62%). This may be due to the higher content of the guluronic acid than that of the monnuronic acid in *P. pavonica* extract, and the similarity of contents of these acids residues in the *S*. *vulgare* extract (9). We suggest that the M/G ratio most probably played a significant role in inhibition of the intracellular replication of the HCV genome *in-vitro*. 

Polysaccharides from the red seaweeds, on the other hand, include different sulfated galactans, sulfated rhamnans or mannans, carrageenans and agars (21). The predominant types of polysaccharides in the studied red seaweeds are sulfated galactan and agar (9). Among the studied brown and red algae, the polysaccharides of the *Laurencia obtuse* extract are the most inhibitor for HCV (82.36% at co-adsorption mode), whereas, the polysaccharides of the *Pterocladia capillacea* extract are the least inhibitor for HCV( 69.85% at co-adsorption mode). This differential inhibition potentiality for the HCV may be due to the high content of sulfate groups with the agarose derivatives in *L. obtuse *(9). The mode of inhibition action in the red seaweed was similar to that of the brown seaweed, on which the co- adsorption has the highest inhibition percentage followed by the pre-adsorption and ending with the post-adsorption mode. The obtained results consistent with previous investigations (e.g. 22) carried out on another enveloped viruses, which suggested that the sulfated polysaccharides in various seaweed species interfer with the attachment of virions to the host cells.

## Conclusion

The present study clarifies action modes of the polysaccharides extracted from brown and red seaweeds on HCV. All seaweeds treatments have the ability to inhibit the HCV. Polyscaccharides from *Laurencia Obtusa *extract are the most inhibitor (82.36% at co-adsorption mode), whereas, those from *Pterocladia capillacea* extract are the least inhibitor (69.85% at co-adsorption mode). This antiviral affects of the seaweed polysaccharides posses promising antiviral properties regarding a broad spectrum of the activity, as well as, the complex mode of the action. Therefore seaweeds represent an interesting candidate for further drug development.
